# LncRNA mediated regulation of aging pathways in *Drosophila melanogaster* during dietary restriction

**DOI:** 10.18632/aging.101062

**Published:** 2016-09-27

**Authors:** Deying Yang, Ting Lian, Jianbo Tu, Uma Gaur, Xueping Mao, Xiaolan Fan, Diyan Li, Ying Li, Mingyao Yang

**Affiliations:** ^1^ Animal Genetic Resources Exploration and Innovation Key Laboratory of Sichuan Province, Sichuan Agricultural University, Chengdu 611130, P.R.China

**Keywords:** aging, lncRNA, Drosophila melanogaster, pathways

## Abstract

Dietary restriction (DR) extends lifespan in many species which is a well-known phenomenon. Long non-coding RNAs (lncRNAs) play an important role in regulation of cell senescence and important age-related signaling pathways. Here, we profiled the lncRNA and mRNA transcriptome of fruit flies at 7 day and 42 day during DR and fully-fed conditions, respectively. In general, 102 differentially expressed lncRNAs and 1406 differentially expressed coding genes were identified. Most informatively we found a large number of differentially expressed lncRNAs and their targets enriched in GO and KEGG analysis. We discovered some new aging related signaling pathways during DR, such as hippo signaling pathway-fly, phototransduction-fly and protein processing in endoplasmic reticulum etc. Novel lncRNAs *XLOC_092363* and *XLOC_166557* are found to be located in 10 kb upstream sequences of *hairy* and *ems* promoters, respectively. Furthermore, tissue specificity of some novel lncRNAs had been analyzed at 7 day of DR in fly head, gut and fat body. Also the silencing of lncRNA *XLOC_076307* resulted in altered expression level of its targets including *Gadd45* (involved in FoxO signaling pathway). Together, the results implicated many lncRNAs closely associated with dietary restriction, which could provide a resource for lncRNA in aging and age-related disease field.

## INTRODUCTION

Dietary restriction (DR) is the best and nongenetic anti-aging strategy without malnutrition, aiming to intervene on environmental factors and reduce the risk of many age-related diseases [1]. DR has been shown to extend lifespan and improve health with the most consistent non-pharmacological intervention during aging in diverse organisms, such as flies [2], yeast [3], nematodes [4] and rodents [5]. These universal effects suggest that there should be some conserved common potential genetic pathways and biochemical processes by virtue of which different organisms delay aging in response to DR [2]. Extensive studies have been devoted to discover such underlying mechanisms of longevity assurance in DR of model organisms including primates (even human) [6]. Previous studies involving whole-genome gene expression analysis have shown that a number of genes and biological pathways differ between organisms fed on DR and normal-diet [2, 7]. A large number of differentially expressed genes (DEGs) are implicated in regulatory mechanism of DR and molecular pathways, such as lipid metabolism, fatty acid metabolism, immune response, and oxidative phosphorylation are identified as being highly responsive to changes in diet composition [8]. Therefore, coding mRNA genes and signaling pathways involved in DR condition have been extensively studied [9]. The long non-coding RNA (lncRNA) is the key regulatory factor which also participates in the modulation of cell senescence and aging-related disease [10]. Pathologies implicating lncRNAs function include cancer [11], neurodegenerative disorders [12], cardiovascular pathologies [13] and metabolic diseases [14]. Some evidences supporting the role of lncRNAs in age-associated molecular processes are emerging recently, for instance, lncRNAs *TERC* and *TERRA* are found to be involved in chromosomal instability and short telomeres, which may contribute to premature senescence and aging [15]. While lncRNAs have significant functions and effect on the course of aging, the number of lncRNAs and their functional implications in anti-aging process are not yet well validated during nutritional interventions especially during DR.

RNA-seq techniques have driven the lncRNAs research in the recent years yielding significant insights in various model species [16]. As the classic model organism, RNA-seq data of coding and non-coding RNA, at different developmental stages, tissue samples, and whole-fly samples treated with environmental per-turbations of *D. melanogaster* have been reported [17, 18]. Interestingly fruit-fly being the ideal model organism for aging related studies can be used to understand the role of lncRNAs, which have not yet been explored for their role in aging. We analyzed RNA-seq data including mRNA and lncRNAs, from a set of DR and fully fed samples at 7 day and 42 day, respectively. Present data provides a deep understanding of the dynamic expression of lncRNAs in *D. melanogaster* transcriptome under DR and fully fed conditions. Our analysis revealed a large number of differentially expressed lncRNAs transcript isoforms and their target genes during DR that spanned most of the genome and can provide valuable insights into aging and age-related disease research field.

## RESULTS

### Dietary restriction extends lifespan

**Figure 1:**
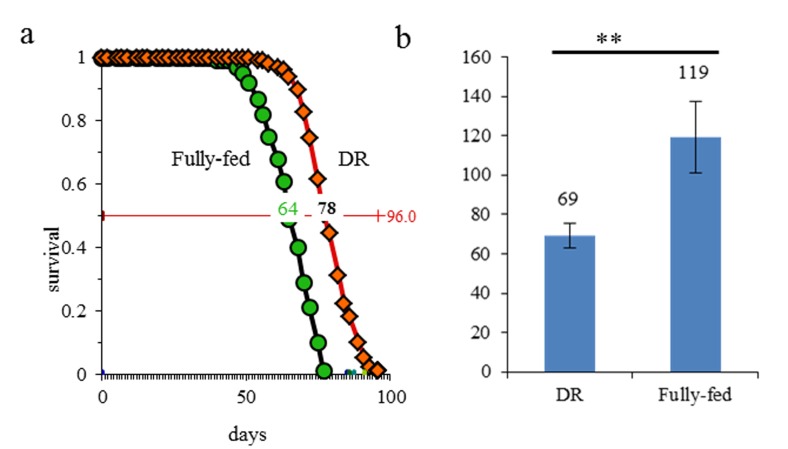
DR extended the lifespan and lowered the egg production in fruit flies (**a**) curve of lifespan; (**b**) the number of eggs produced; **, *p*≤0.01.

Similar to our previous report [19], the fruit fly females, on DR food had median lifespans extended by 21.88% in comparison to fully fed flies in the present study (Fig. 1a). Fecundity of fruit flies in DR group became lower than in fully fed group (*P*<0.01, Fig. 1b). Flies at day 7 have been considered as young flies in many previous reports [20, 21] and the flies in the present study began to die at day 42. Thus, 7 day and 42 day are the most appropriate time points for carrying out aging specific analysis involving expression patterns of transcriptional dynamics through lncRNA analysis.

### Transcriptome profiles of DR and fully fed flies

**Figure 2:**
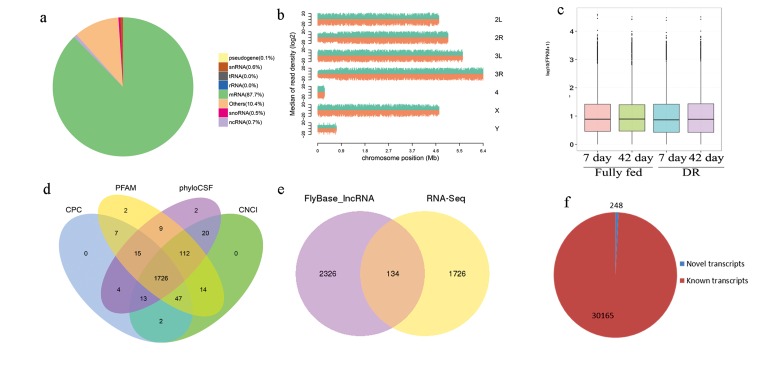
Overall expression profile in RNA-seq (a) mapping to fruit fly genome; (b) reads density in chromosomes; (c) FPKM distribution of mRNA and lncRNA; (d) 1726 novel lncRNAs were obtained after four programs filtering; (e) when compared to 2460 known lncRNAs sequences in FlyBase (Dmel_Release_6), 134 non-redundant known lncRNAs and 1726 non-redundant novel lncRNAs were found; (f) When compared to known mRNA sequences in FlyBase, 30165 known and 248 novel transcripts were found.

A total of 86.78 GB of raw sequence data was produced yielding 7.26 billion strand-specific raw reads (Table S1). Value of Q20, Q30 and GC content were not significantly different among samples. After quality check, 95.79% of clean reads were obtained and were aligned to the *D. melanogaster* genome from FlyBase (Dmel_Release_6, http://FlyBase.org/), assigning 87.7% reads to the mRNA sequences (Fig. 2a). The read density (log2) was distributed mainly in 2L, 2R, 3L, 3R and X chromosomes (Fig. 2b). FPKM (fragments per kilobase of transcript sequence per millions base pairs sequenced) distribution was not significantly different in expression levels between lncRNAs and mRNA in DR and fully fed flies (*P*≤0.05, Fig. 2c).

In total, 3826 redundant lncRNA transcripts and 30413 redundant mRNA transcripts were identified in this study. After BLASTing with FlyBase lncRNA (Dmel_Release_6), these 3826 transcripts corresponded to a total of 134 non-redundant known lncRNAs (Dataset S1) and 1726 non-redundant novel lncRNA (Dataset S2, Fig. 2d and 2e). In those novel lncRNAs, the number of large intergenic noncoding RNA, intronic transcript lncRNAs and antisense lncRNAs were 821, 695 and 210, respectively. A total of 248 non-redundant novel mRNA transcripts (Dataset S3, Fig. 2f) were recognized when 30413 mRNA transcripts were BLASTed with *D. melanogaster* genome from FlyBase, and the remaining 30165 transcripts aligned to 13917 genes.

### Differentially expressed genes analysis

**Figure 3:**
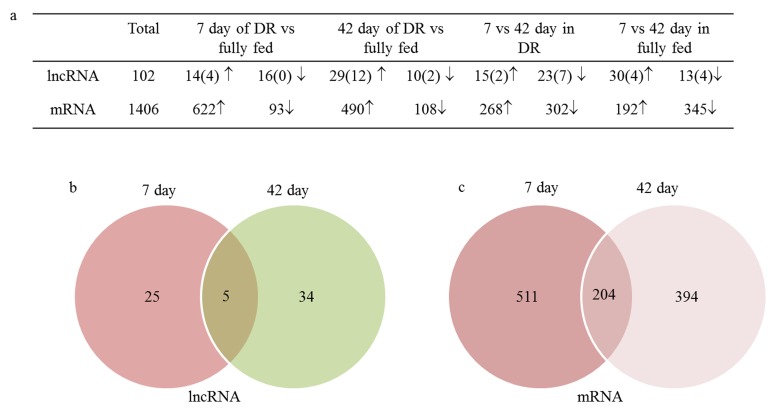
102 differentially expressed lncRNA and 1406 differentially expressed coding genes were identified in this study. (**a**) The number of DEGs in different comparative groups (parentheses presented the number of known lncRNA); (**b**) Common differentially expressed lncRNA in both 7 day and 42 day between DR and fully fed flies; (**c**) Common differentially expressed mRNA in both 7 day and 42 day between DR and fully fed flies; ↑, upregulation; ↓, downregulation.

Differentially expressed genes (DEGs) had been analyzed between four comparative groups (Dataset S4), including 7 day DR vs 7 day fully fed, 42 day DR vs 42 day fully fed, 7 day DR vs 42 day DR, 7 day fully fed vs 42 day fully fed. In total, 102 differentially expressed lncRNAs (25 known and 77 novels) and 1406 coding genes were identified between these four comparative groups. A total of 719 differentially expressed transcripts were the most abundant as measured at 7 day between DR and fully fed group, including 30 lncRNAs and 689 coding mRNA genes. Also 39 differentially expressed lncRNAs and 571 differentially expressed coding genes were present at 42 day of DR when compared to fully fed group (Fig. 3a). Out of those differentially expressed transcripts, five common differentially expressed lncRNAs and 204 common coding genes appeared at both 7 day and 42 day between DR and fully fed groups (Fig. 3b, 3c).

Among five differentially expressed lncRNAs, *CR31781* and *XLOC_014901* were up-regulated while *XLOC_189941* was down-regulated in DR when compared to fully fed group at both time frames. Another two lncRNAs (*XLOC_189909* and *XLOC_228709*) were down-regulated at 7 day and up-regulated at 42 day in DR when compared to fully fed group.

In order to validate the RNA-seq analysis finding, we randomly selected 28 differentially expressed lncRNAs and 61 mRNA coding genes, and analyzed their expression using quantitative real-time PCR (qPCR) analysis. All the genes were having expression levels in concordance with the RNA-seq results in different comparison groups with varying levels of significance (*P*≤0.05) (Dataset S5).

### Known lncRNA

When compared to the known lncRNAs existing in FlyBase (Dmel_Release_6), 134 known lncRNAs in our study were detected (Dataset S1). Of 134 annotated lncRNAs, 25 differentially expressed lncRNAs in four different comparisons (7 day DR vs 7 day fully fed, 42 day DR vs 42 day fully fed, 7 day DR vs 42 day DR, 7 day fully fed vs 42 day fully fed). Furthermore, 4 and 14 known lncRNAs displayed differential expression at the 7 day and 42 day when compared between DR and fully fed groups, respectively. Moreover, out of a total of 134 annotated lncRNAs only 11 lncRNAs obtained 22 annotations from GO enrichment. *CR31781* was the only one differentially expressed lncRNA which got GO annotation and was further validated by qPCR result. A total of 15 out of 22 annotations belonged to lncRNA *CR31781*, such as lateral inhibition, cell-cell signaling involved in cell fate commitment, extracellular space, extracellular region part and cellular component, etc.

### Novel lncRNA

We predicted the targets of novel lncRNAs, and then analyzed the GO (Dataset S6) and KEGG enrichment (Dataset S7) of targets (Dataset S8 and Dataset S9). In most GO enrichment annotations, 17 non-redundant differentially expressed mRNA genes were the targets of 13 differentially expressed lncRNAs (Table 1). Six differentially expressed coding genes were the target of six differentially expressed lncRNAs and expressed during both 7 day and 42 day in DR when compared to fully fed fruit flies, and were found to be enriched in carboxypeptidase activity, cellular component morphogenesis, heme binding, iron ion binding and serine-type endopeptidase activity. In all the comparison groups, nine differentially expressed target genes related to fruit fly aging, which has resulted in lethal (including *CG9733*, *SPH93*, *Hsc70-1*, *Cyp4p3*, *IscU* homolog genes) and long lived (including *Socs36E* genes) phenotypes under RNAi system.

**Table 1:** GO annotations of differentially expressed lncRNA and their targets in DR group when compared to fully fed group

lncRNA(action mode, corrected p-value)	Targets(corrected p-value)	GO annotations
7 day DR vs 7 day fully fed				
XLOC_052958(cis, 0.00715)↓	CG12374(0.00005)↑	Carboxypeptidase activity
XLOC_166557(trans, 0.00425)↓	globin 1(0.0214)↑	Heme binding/Iron ion binding
XLOC_009418(trans, 0.0322)↑	CG6048(0.0496)↑	Serine-type endopeptidase activity
42 day DR vs 42 day fully fed		
XLOC_001163(trans, 0.0171)↑	Hsc70-1(0.0045)↑	Cellular component morphogenesis
XLOC_196039(trans, 0.0028)↑	Cyp28d2(0.00835)↑	Heme binding
XLOC_009798(trans, 0.01205)↑	IscU homolog(0.00395)↑	Iron ion binding
7 day DR vs 42 day DR		
XLOC_002137(trans, 0.02335)↓	Hsc70-3(0.0097)↓	Cell morphogenesis/Cellular component morphogenesis
	CG9733(0.00005)↓	Serine-type endopeptidase activity
XLOC_076307(trans, 0.03315)↓	Hsp70Bc(0.00005)↓	Cell morphogenesis/Cellular component morphogenesis/Glycosaminoglycan catabolic process/Glycosaminoglycan metabolic process/Peptidoglycan catabolic process/Peptidoglycan metabolic process/Serine-type endopeptidase activity
	Socs36E(0.00005)↓	Cell morphogenesis/Cellular component morphogenesis
	CG10041(0.0489)↓	Serine-type endopeptidase activity
		
XLOC_000246(trans, 0.01425)↓	CG15046(0.0405)↓	Serine-type endopeptidase activity
XLOC_071173(trans, 0.0129)↓	SPH93(0.00005) ↓	Serine-type endopeptidase activity
XLOC_066439(cis, 0.0113)↓	Cyp6w1(0.0103)↓	Heme binding/Iron ion binding
XLOC_009798(trans, 0.0034)↓	Cyp4p3(0.01165)↓ CG10041(0.0489)↓ IscU homolog(0.0115)↓	Heme binding/Iron ion binding Serine-type endopeptidase activity Iron ion binding
7 day fully fed vs 42 day fully fed		
XLOC_052958(cis, 0.0087)↑	CG12374(0.0001)↑	Carboxypeptidase activity
XLOC_067962(cis, 0.0097)↑	PGRP-SC1a(0.00005)↑	Carboxypeptidase activity/ Glycosaminoglycan catabolic process/ Glycosaminoglycan metabolic process/Peptidoglycan catabolic process
XLOC_151622(cis, 0.00635)↑	CG33970(0.0346)↓	Iron ion binding

Note: ↑, up-regulation; ↓, down-regulation.

In most significant KEGG pathway enrichment (Dataset S7), 27 differentially expressed lncRNAs and 44 differentially expressed targets were found to be involved in 38 pathways out of which 21 targets were related to fly aging. Most of these were important pathways affecting senescence, such as mTOR, FoxO and Wnt signaling pathway. At 7 day and 42 day, 11 of the 26 genes in the mTOR pathway were present in the RNA-seq data, but, just *inac* gene was upregulated as the target of lncRNA *XLOC_056059* at the 42 day in DR in comparison to 42 day in fully fed. Five lncRNAs and three targets existed in FoxO signaling pathway, and were found upregulated at 42 day in DR when compared to fully fed flies at same day (Table 2). Especially, target genes involved in signaling pathways of biosynthesis of unsaturated fatty acids and fatty acid metabolism were up-regulated in DR group in comparison to fully fed, including *CG9743* (as the target of *XLOC_201255*), *CG15531*, *CG9743*, *CG15531* and *FASN2* at the 7 day, *Acsl* and *CG3961* at the 42 day.

**Table 2:** Novle lncRNAs and mRNA genes involved in five KEGG pathways during DR

KEGG pathway	lncRNA (comparable group and correct pvalue, action mode)	mRNA genes (comparable group and correct pvalue)
FoxO signaling pathway	XLOC_000246(A2↑-B2 0.044, A1↓-A2 0.01425, trans) XLOC_076307(A1↑-A2 0.03315, trans)	Gadd45 (A2↑-B2 0.01075, A1↓-A2 0.014)
	XLOC_000043(A2↑-B2 0.0136, A1↓-A2 0.01345, trans)	Pepck(A2↑-B2 0.00005, A1↓-A2 0.00005)
	XLOC_221562(A2↑­-B2 0.00275, trans) XLOC_001163(A2↑­-B2 0.0171, trans)	Egfr(A2↑-B2 0.0204)
Hippo signaling pathway-fly	XLOC_002137(A1↓-A2 0.02335, trans)	Upd2(A1↑-A2 0.0043)
	XLOC_002137(A1↓-A2 0.02335, trans)	Upd3(A1↓-A2 0.01585)
	XLOC_186922(A1↓-B1 0.00785, cis)	Zyx(A1↓-B1 0.046)
	XLOC_000246(A1↓-A2 0.01425, trans) XLOC_000236(A1↓-A2 0.00555, trans) XLOC_009798(A1↓-A2 0.0034, trans) XLOC_000043(A1↓-A2 0.01345, trans)	baz (A1↓-A2 0.03595)
	XLOC_160411(B1↓-B2 0.0048, trans)	wg(B1↓-B2 0.00905)
Phototransduction-fly	XLOC_092363(A1↓-B1 0.00885, B1↑-B2 0.0075,cis)	Arr2(A1↑-B1 0.00315, B1↑-B2 0.0481)
Protein processing in endoplasmic reticulum	XLOC_076307(A1↓-A2 0.03315, trans)	Hsp70Bc(A1↓-A2 0.00005)
	XLOC_001163(A2↑-B2 0.0171, trans)	Hsc70-1 (A2↑-B2 0.0045)
	XLOC_072226(A1↓-B1 0.00885, trans)	CG8974(A1↑-B1 0.01995)
	XLOC_002137(A1↓-A2 0.02335, trans)	Hsc70-3(A1↓-A2 0.02335)
	XLOC_000043(A2↑-B2 0.0136, A1↓-A2 0.01345, trans) XLOC_009798(A2↑-B2 0.01205, A1↓-A2 0.0034, trans) XLOC_000236(A1↓-A2 0.00555, A2↑-B2 0.00955, trans)	l(2)efl(A1↓-A2 0.0108, A2↓-B2 0.01715)
Metabolism of xenobiotics by cytochrome P450	XLOC_002137(A1↓-A2 0.02335, trans)	GstD5(A1↓-A2 0.00005)
	XLOC_000246(A2↑-B2 0.044, A1↓-A2 0.01425, trans) XLOC_002137(A1↓-A2 0.02335, trans)	GstD10 (A2↑-B2 0.0138, A1↓-A2 0.00175)
	XLOC_201255(A1↑-B1 0.00515, trans)	GstE12 (A1↑-B1 0.0495)
	XLOC_010702(A1↑-A2 0.0023, trans)	CG17323(A1↑-A2 0.0108)
	XLOC_002137(A1↓-A2 0.02335, trans)	GstE7 (A1↓-A2 0.00005), CG5999(A1↓-A2 0.00135)
	XLOC_106174(A1↑­-B1 0.02315, trans)	Ugt35b ( A1↑-B1 0.00015)
Glutathione metabolism	XLOC_002137(A1↓-A2 0.02335, trans)	GstD10(A1↓-A2 0.00175), GstD5(A1↓-A2 0.00005), GstE7(A1↓-A2 0.00005)
	XLOC_201255(A1↑-B1, trans)	GstE12(A1↑-B1 0.0495)

Note: A1, 7 day in DR; A2, 42 day in DR; B1, 7 day in fully fed; B2, 42 day in fully fed; ↑, up-regulation; ↓, down-regulation.

In the present study, we have characterized three novel signaling pathways that may be related to aging, which are, hippo signaling pathway-fly, phototransduction-fly and protein processing in endoplasmic reticulum (Table 2). In hippo signaling pathway-fly 11 differentially expressed coding genes were involved and, three and five genes displayed differential expression at the 7 day and 42 day in DR when compared to fully fed flies, respectively, and five coding genes were the targets of seven novel lncRNAs. In phototransduction-fly signaling pathway eight related coding genes were detected and showed differential expression between various comparison groups in DR and fully fed condition. Novel lncRNA *XLOC_092363* and respective target *Arr2* were involved on phototransduction-fly signaling pathway. Furthermore, in endoplasmic reticulum pathway 15 differentially expressed coding genes were found in protein processing and 11 of these were related to aging and five genes were the targets of 7 novel lncRNAs. Interestingly enough, 9 of 15 differentially expressed coding genes belonged to heat shock protein gene families.

Besides, 17 DEGs and 14 DEGs presented in metabolism of xenobiotics by cytochrome P450 and glutathione metabolism pathway, respectively, which were up-regulated in various comparison groups in DR when compared to fully fed condition (Table 2). Out of these, 10 of 17 DEGs and 11 of 14 DEGs belonged to glutathione S transferase, and there are some common glutathione S transferase genes between both pathways such as GstD5, GstD10, GstD6, GstE1, GstE2, GstE3, GstE4, GstE7 and GstE12. Meanwhile seven targets of five novel lncRNAs and four targets of two novel lncRNAs were found in cytochrome P450 and glutathione metabolism pathway, respectively.

### Regulatory elements of lncRNA

The promoter positions in genome of fruit fly were first confirmed according to *Drosophila* Core Promoter Database, followed by analysis of lncRNA located in 10kb range (90% identification accuracy and 90% coverage, Dataset S10). Forty four novels and one known lncRNA in our study were found in 10 kb range of 38 promoters. Among 45 lncRNAs, 10 lncRNAs and 12 promoters were present at X chromosome, while one lncRNA and one promoter were present at 2L chromosome. Furthermore, 4 lncRNAs and 4 promoters had the overlapping zones, including *XLOC_067699* (promoter *lcp4*, overlap 92bp), *XLOC_112986* (promoter *primase*, overlap 92bp), *XLOC_113215* (promoter *s15*, overlap 92bp) and *XLOC_220979* (promoter *G6pd*, overlap 94bp).

**Figure 4:**
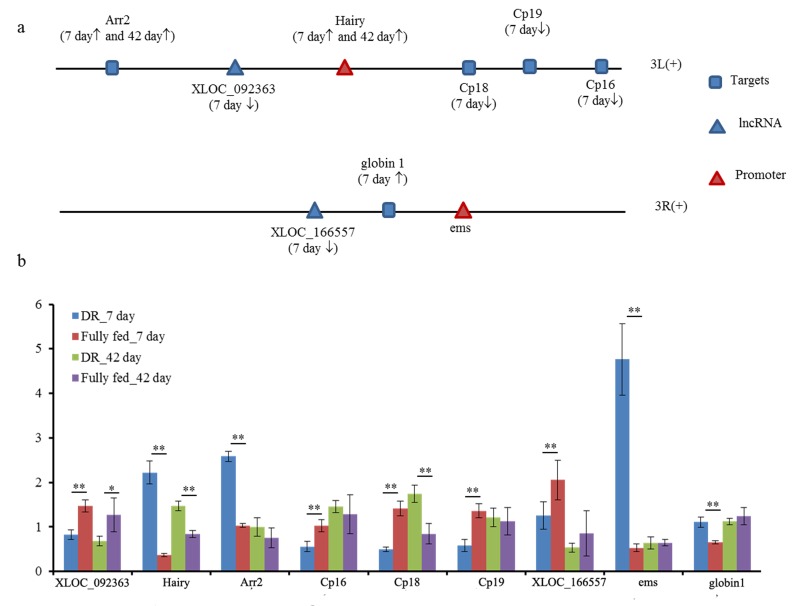
Two promoters associated lncRNA *XLOC_092363* and *XLOC_166557* in 10 kb range (**a**) the positional relationship of two differentially expressed lncRNA and targets in chromosome of fruit fly (↑, up-regulated in DR when compared to fully fed group in RNA-seq data; ↓, down-regulated in DR when compared to fully fed group in RNA-seq profile); (**b**) expression level of two promoters, lncRNA and their targets between DR and fully fed flies by qPCR. **, *P*≤0.01; *, *P*≤0.05.

Interestingly enough, two novel lncRNAs (*XLOC_092363* and *XLOC_166557*) differentially expressed at the 7 day in DR group when compared to fully fed group. lncRNA *XLOC_092363* was located in promoter hairy in the up-stream of sense strand of 3L chromosome. Five differentially expressed targets of *XLOC_092363* were also located in 3L chromosome through *cis*-acting mode (Fig. 4a). It's noteworthy that *hairy* gene as associated promoter and as target of *XLOC_092363*, differentially expressed at 7 day and 42 day of DR in comparison to fully fed flies. Target *arr2* has the same tendency of being differentially expressed with promoter *hairy* gene. Other three targets *Cp16*, *Cp18* and *Cp19* differentially expressed at the 7 day in DR when compared to fully fed group, and their molecular function was being the structural constituent of chorion. In addition, promoter ems sited in the down-stream of *XLOC_166557* in sense strand of 3R chromosome is involved in heme binding and iron ion binding of GO annotations (Fig. 4a). The target of *XLOC_166557* was globin 1 by *cis*-acting mode, and *XLOC_166557* was found to be down-regulated while globin 1 was up-regulated in DR when compared to fully fed flies. The qPCR results were consistent with RNA-seq data up to certain extent (Dataset S3), and revealed differential expression between DR and fully fed flies (Fig. 4b).

### Tissue specificity of lncRNA

Differentially expressed lncRNA and mRNA in this study were BLASTed with the transcripts from *D. melanogaster* from cultured cell lines, dissected organ systems and under environmental perturbations and 5 known lncRNAs from the present study showed the tissue specificity (Dataset S11). In five lncRNAs, *CR31781* and target *CG31810* (*cis*-acting mode) were upregulated in DR group when compared to fully-fed group, which were mainly expressed in head, carcass, digestive system, central nervous system and under environmental perturbations (including caffeine, paraquat, Cd, Zn, Cu) in fruit fly. Another target *CG13284* was seen upregulated at the 42 day when compared between DR and fully-fed groups. The molecular function of *CG31810* and *CG13284* is described with steroid dehydrogenase activity. Besides, lncRNA *CR43641* (up-regulated at the 7 day when compared to fully fed group) along with the differentially expressed targets *Dip-B*, *tal-AA* and *CG34383* (*cis*-acting mode), were highly expressed in fat or fat body. The remaining lncRNA (*CR43148*, *CR40469* and *CR44095*) and their differentially expressed targets did not have the obviously same tissue specificity.

**Figure 5:**
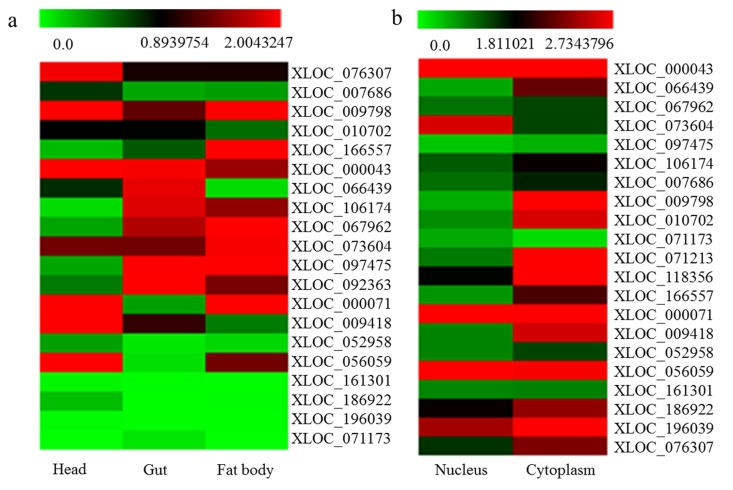
Tissue specificity of differentially expressed lncRNA (**a**) the tissue specificity of 20 differentially expressed lncRNA at 7 day under DR condition had been analyzed in gut, head, and fat body of flies; (**b**) 21 differentially expressed lncRNA whether located in cytoplasm or nucleus of Schneider's line 2 cell were detected.

A total of 20 novel differentially expressed lncRNAs at 7 day under DR condition were randomly selected for analysis in fly head, gut, and fat body using qPCR to determine the tissue specificity (Fig. 5a, primers in Table S2). The results showed that lncRNA *XLOC_066439* were at significantly higher expression level in gut compared to head and fat body (*P*≤0.05). Also the expression levels of *XLOC_07630*7 and *XLOC_009418* in head were significantly higher than that of gut and fat body (*P*≤0.05). *XLOC_166557* was mainly expressed in fatbody of fruit flies. Tissue specificity of several lncRNA was consistent with their targets (Table S3), such as *XLOC_076307* (*Socs36E*, head), *XLOC_166557* (*globin 1*, fatbody), *XLOC_067962* (*PGRP-SC1a*, *PGRP-SC2* and *CG8740*, gut and fatbody). On the other hand, 21 differentially expressed lncRNAs were identified in cytoplasm or nucleus of Schneider's line 2 cells by qPCR (Fig. 5b). Among these lncRNA, *XLOC_073604* was specially expressed in S2 cell's nucleus. LncRNA *XLOC_009798*, *XLOC_010702*, *XLOC_071213*, *XLOC_118356*, *XLOC_009418* and *XLOC_076307* were particularly located in S2 cell's cytoplasm. We also found that lncRNA *XLOC_000071*, *XLOC_056059* and *XLOC_196039* were expressed in S2 cell's nucleus and cytoplasm.

### Inhibition of a novel lncRNA expression in fly S2 cell

**Figure 6:**
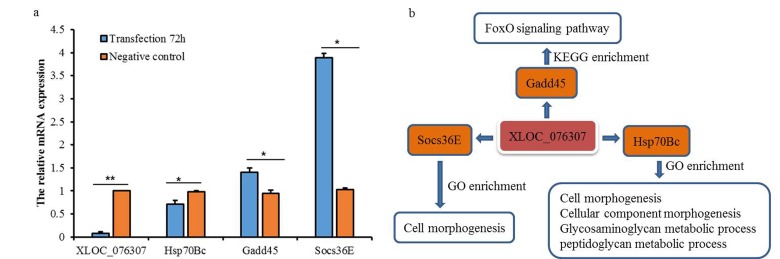
Expression levels of three targets after 72h of transfection XLOC_076307 siRNA and function enrichment (**a**) expression level of lncRNA XLOC_076307 was significantly down regulated with target Hsp70Bc down-regulation and Gadd45 and Socs36E up-regulation; (**b**) function enrichment (including GO and KEGG) of XLOC_076307 and its targets.

A novel lncRNA, targets among other few, the *Gadd45* gene which is involved in FoxO signaling pathway (Fig. 6). In order to understand the role of this novel lncRNA *XLOC_076307*, we silenced it in fruit fly S2 cells by small interfering RNA (siRNA). The results showed that the expression level of *XLOC_076307* and its target *Hsp70Bc* decreased significantly (*P*≤0.01), while targets *Gadd45* and *Socs36E* showed significant increases after 72h of transfection, confirming the association between lncRNA and its predicted targets through *trans* action model.

## DISCUSSION

Long non-coding RNAs make up the majority of the mammalian transcriptome, many of which are emerging as regulators of genome expression/stability and modulators of cell senescence [22, 23]. Previous studies have shown that lncRNAs are involved in aging process and age-related diseases [24, 25]. Although DR is the best-studied intervention known to delay aging and extend lifespan in evolutionarily distant organisms ranging from fruit flies to mammals in the laboratory, the molecular mechanisms of DR are still elusive. In our study, we analyzed lncRNA and mRNA gene expression patterns of transcriptional dynamics in fruit flies at the 7 day and 42 day during DR and fully-fed conditions, respectively, and identified the differentially expressed lncRNAs and their target genes and revealed their possible regulatory roles in DR process.

A large number of candidate lncRNAs have been identified in *Drosophila* during different developmental stages, dissected tissue samples, cultured cell lines and under environmental perturbations [17, 25, 26]. In this study, we found only 134 known lncRNAs when compared to 2460 annotated lncRNAs from FlyBase (Dmel_Release_6). The reason may be the technical differences between ours and previous studies. Also the lncRNAs at the time of DR have not been identified before and thus, the number of novel lncRNAs (1726) was far more than known lncRNA (134) related to DR, demonstrating that DR impact on lncRNAs remains largely unexplored.

### LncRNA in known aging pathways

Upon comparison of aging-related signaling pathways with previously published data on fruit flies [26], we found differentially expressed coding genes as the targets of lncRNA which were enriched in signaling pathways, such as mTOR, FoxO and Wnt signaling pathway, biosynthesis of unsaturated fatty acids, and fatty acid metabolism. Especially in FoxO signaling pathway, three (*Gadd45, Pepck, Egfr*) of seven differentially expressed coding genes were found to be up-regulated at the 42 day of DR when compared to fully fed flies as the targets of novel lncRNA (*XLOC_000246, XLOC_076307, XLOC_000043, XLOC_221562* and *XLOC_001163*). *Gadd45* and *Egfr* had been reported to be involved in *Drosophila* senescence [27, 28]. Adipose tissue is a major source of energy, which declines in old age [23, 29]. In our study, DR activates the level of biosynthesis of unsaturated fatty acids and fatty acid metabolism and may rescue the declining stored fat with advancing age to extend healthspan of *Drosophila* flies. CG9743 (up-regulated at the 7 day of DR versus fully fed flies) as the target of novel differentially expressed lncRNA XLOC_201255 participates in lipid metabolic process [30], such as unsaturated fatty acids and fatty acid metabolism, suggesting a possible involvement in regulation of lipid metabolic process. In this study, 1 gene (inac gene) out of 11 mTOR pathway genes were found to be differentially expressed, and the remaining genes were not significantly different between any of the comparative groups. This result is consistent with what has been previously reported [2]. We presume that the effects of DR on the mTOR pathway genes may occur post-transcriptionally and could not be detected in RNA-seq data. From those results, DR alters the expressing profile of mRNA and lncRNA to activate of the conserved nutrient-signaling pathways.

### lncRNA in potential novel aging pathways

Besides known aging-related signaling pathways, we also identified novel pathways which might be related to senescence in DR process. In present study, 11 DEGs were detected which were involved in Hippo pathway and nine of these had been demonstrated to be related to *Drosophila* aging, such as *Upd2*, *Upd3*, *Zyx*, *Pp2A-29B*, *baz*, *wg*, etc. The Hippo pathway is an evolutionarily conserved and pleiotropic mediator of growth control, cell fate decisions and stem cell identity, cell type specification and differentiation during development [31], and, Hippo pathway components also mediated crosstalk with other pathways, for example Wnt, TGF and BMP pathways[32], and also five targets of seven lncRNAs were present in Hippo pathway in the present study.

Phototransduction-fly was another interesting pathway, and had eight DEGs (27.57% of the total 28 coding genes) upregulated during DR when compared to fully fed group in present study. Transient receptor potential (*trp*) was the essential element of the transduction cascade [33], which was upregulated at the 7 day and 42 day during DR in comparison to fully fed flies in our study. Furthermore, the differentially expressed *Arr2* and *inaC* genes were the targets of differentially expressed lncRNA *XLOC_092363* and *XLOC_056059*, respectively. These results indicated DR to be the pleiotropic mediator of *Drosophila* aging for affecting various physiological processes. LncRNA may also take part in regulation of these similar pathways.

### lncRNA and its relation with gene promoters

Previous studies have documented the important connection of lncRNA to promoter. Promoter region could be transcribed into lncRNA in quiescent cells to block DHFR gene expression through both direct interactions of the RNA and promoter-specific interference [34]. In our study, we found that 4 lncRNA and 4 promoters had the overlapping zones, which illustrates that these lncRNAs may be transcribed from corresponding promoters.

Another study showed lncRNA *HSR1* (heat shock RNA-1) was required for *HSF1* (heat-shock transcription factor 1) activation, and the activated *HSF1* affect the promoter of related genes to open the transcription in mammalian cells[35]. In this study, we found two differentially expressed lncRNA had the position correlation with promoters. As promoter and target of lncRNA *XLOC_092363*, hairy gene up-regulation in DR had been proven to be a lethal factor after mutagen treatment [36]. Furthermore, *Drosophila sirt1* is required for heterochromatic silencing and euchromatic *hairy*/E(Spl) bHLH repression in segmentation and sex determination[36], which is a key member of the sirtuin family of proteins and mammalian homolog of the yeast silent information regulator protein that influence lifespan in several model organisms[37]. *XLOC_092363* and targets *Cp16*, *Cp18* and *Cp19* were down-regulated at 7 day of DR while promoter hairy was up-regulated. *Cp16*, *Cp18* and *Cp19* are involved in biological process of multicellular organismal development and eggshell chorion assembly[38]. We also observed a decrease in egg production in DR flies suggesting that promoter hairy may have special correlation with lncRNA *XLOC_092363* and downstream targets to affect fecundity in DR flies.

Another lncRNA *XLOC_166557* was located in upstream of promoter ems and the expression level of ems was up-regulated at the 7 day during DR when compared to fully fed flies. Its target globin1 located in downstream participated in biological process of oxygen transport [39]. In summary, these results demonstrate that differentially expressed lncRNA and promoters may play a role in DR for extending lifespan *in Drosophila*.

### Tissue specificity of lncRNA

In this study, two differentially expressed annotated lncRNA (*CR31781* and *CR43641*) and its targets have the consistent tissue specificity. *CR31781* presented specific expression at the 42 day of DR when compared to fully fed flies, and its molecular function is lateral inhibition in cell fate determination [40]. *CR31781* and two targets had the similar tissue specificity in carcass from mated female after 4 days [17]. The previous studies reported the variable expression level of *CR31781* (presenting up-regulation) in myopathic lamin mutations and lifespan extension of flies over-expressing a small mitochondrial chaperone *Hsp22* [41]. Furthermore, its potential targets *CG31810* and *CG13284* are involved in steroid metabolic process, had steroid dehydrogenase activity and upregulated in DR. *CG13284* also participated in negative regulation of cell proliferation and phagocytosis with relevant senescence [42]. These observations suggest that annotated lncRNA *CR31781* and its predicted targets may play an important role in DR, and the specific mechanism of action should be studied in the future research.

Another lncRNA *CR43641* and targets *Dip-B*, *tal-AA* and *CG34383* were highly expressed in *Drosophila* fat body, a major immune organ, that undergoes immuno-senescence and mounts strong systemic inflammation leading to dysregulation of immune deficiency signaling in the midgut of old flies [43]. RNAi system of target *CG34383* (Scer\GAL4pnr-MD237) had been a lethal factor before end of pupal stage [40] related to *Drosophila* aging. Target *Dip-B* participated in proteolysis process while *tal-AA* took part in biological process of actin filament organization and morpho-genesis of an epithelium [44]. Based on these findings, we would speculate that lncRNA *CR43641* and its predicted targets may have the positive function in enhancing the immune system and anti-aging in DR conditions.

### lncRNA *XLOC_076307* relationship with its targets

We have identified in the Schneider's line 2 cells, the novel lncRNA *XLOC_076307*, which was hypothesized to possibly take part in cell morphogenesis, cellular component morphogenesis, glycosaminoglycan metabolic process, peptidoglycan metabolic process, spliceosome and FoxO signaling pathway according to its potential targets (*Hsp70Bc*, *Socs36E* and *Gadd45*), and was found mainly located in flies head and S2 cell cytoplasm. Loss of *Hsp70* in *Drosophila* is pleiotropic, with effects on thermotolerance, recovery from heat shock, neurodegeneration and aging defects [45]. Also the target *Hsp70Bc* was related to heat shock-mediated polytene chromosome puffing [45]. Another target *Socs36E* played a critical role in a genetic circuit that establishes a boundary between the motile border cell cluster and its non-invasive epithelial neighbors through STAT attenuation [46]. Furthermore, phenotypes Socs36E^Scer\UAS.P\T.cCa^ could reduce death in some flies dying during pharate adult stage [46]. The over-expression of target *Gadd45* in the nervous system of *D. melanogaster* increased the lifespan [47]. Expression level of three targets changed after 72h of transfection of *XLOC_076307* siRNA in our study. Thus, we hypothesize that *XLOC_076307* may play a role in *Drosophila* aging.

## MATERIALS AND METHODS

### Sample collection and preparation

The wild-type stock (Dahomey) were reared at 25°C on a 12-hour on/off light cycle at 50% humidity. Female flies were sorted in two groups which were given DR and fully fed treatment [48]. Median lifespan was calculated as the mean life span of the longest surviving 50% of the population. Egg count was measured according to Emran et al [19]. Ten vials (100 females) of flies were randomly collected for RNA-seq at the 7 day and 42 day in experimental groups (including DR and fully fed group), respectively. A total of eight samples (two biological replicates at day 7 and 42 for each treatment group) were chosen for paired-end (PE read length of 100bp) sequencing using Hiseq2000.

### RNA isolation, library preparation, sequencing and lncRNA prediction

Total RNA was extracted using TRIzol Reagent (Invitrogen, CA), and treated with DNase and purified on an RNAeasy column (Qiagen). The quality, quantity and integrity were measured using standard laboratory procedures. Subsequently, sequencing libraries were generated using the rRNA-depleted RNA by NEBNext® Ultra™ Directional RNA Library Prep Kit for Illumina® (New England Biolabs, USA) following manufacturer's recommendations and library quality was assessed on the Agilent Bioanalyzer 2100 system. After cluster generation, the libraries were sequenced on an Illumina Hiseq 2000 platform (provided by Novogene Co. China) and 100 bp paired-end reads were generated. After removing low quality reads (QV\20) and trimming the adapters, paired-end clean reads were aligned to the fly reference genome using TopHat v2.0.9 [49].

Cuffcompare software [49] was used to combine the transcripts, which at least existed in two samples. The ≥200bp transcripts were selected and the number of extrons was ≥1. Those transcripts were BLASTed (evalue=1e-10) to FlyBase dataset. The mapped transcripts were directly described as known lncRNAs or mRNA genes. The remaining transcripts were BLASTed (evalue=1e-10) to non-mRNA transcripts, such as rRNA, tRNA, snRNA, snoRNA, pre-miRNA, pseudogenes. And, the similar or same transcripts were filtered out. Then, CNCI (Coding-Non-Coding-Index) (v2) profiles [50], CPC (Coding Potential Calculator) (0.9-r2) [51], Pfam Scan (v1.3) [52], and PhyloCSF (phylogenetic codon substitution frequency) (v20121028) [53] were performed to analyse the coding potential of transcripts. Transcripts predicted with coding potential by either/all of the four tools mentioned above, were filtered out, and those without coding potential were our candidate set of novel lncRNAs. The non-coding transcripts were BLASTed to mRNA in FlyBase dataset to screen the lincRNA, intronic lncRNA, anti-sense lncRNA trans-cripts by Cuffcompare according to class_code (http://cufflinks.cbcb.umd.edu/manual.html#class_codes) [54] and the transcripts with coding potential were the novel mRNA transcripts.

### Quantification of gene expression level

Cuffdiff (v2.1.1) [49] was used to calculate FPKMs (expected number of fragments per kilobase of transcript sequence per millions base pairs sequenced) of both lncRNAs and coding genes in each sample. Gene FPKMs were computed by summing up the FPKMs of transcripts in each gene group.

### Target gene prediction

LncRNAs have regulatory roles in gene expression at both the transcriptional and post-transcriptional levels in diverse cellular contexts and biological processes. lncRNAs are responsible for nuclear structure integrity, and can regulate the expression of either nearby genes (acting in cis in the nucleus) or genes elsewhere in cells (acting in trans in the nucleus or cytoplasm) by interacting with proteins, RNAs, and DNAs [55, 56].*Cis* acting lncRNAs are those acting on neighboring target genes [55]. We searched coding genes 10k/100k upstream and downstream of lncRNAs and then analyzed their function next. *Trans* acting targets were identified by expression levels of both lncRNAs and mRNA transcripts [56]. While there were eight samples, we calculated the expressed correlation between lncRNAs and coding genes with custom scripts; otherwise, we clustered the genes from different samples with WGCNA [57] to search common expression modules and then analyzed their function through functional enrichment analysis.

### Differential gene expression analysis

Cuffdiff (v2.1.1) [49] was used to calculate FPKMs and p value of both lncRNAs and coding genes in each sample. A differential expression correct p value (using BH orthosis)≤0.05 and fold change > 1.5 assigned as differentially expressed in four different comparisons (7 day DR vs fully fed, 42 day DR vs fully fed, 7 day DR vs 42 day DR, 7 day fully fed vs 42 day fully fed). qRT-PCR was used to validate 28 differentially expressed lncRNAs and corresponding 61 targets using eight samples from two biological duplicates including DR and fully fed groups at the 7 day and 42 day, and the relative gene expression level was calculated according to Livak and Schmittgen methods [58]. All custom primers were purchased from Sangon Biotech, and the primers of internal reference and identified transcripts in the qPCR were presented in Table S3. The SYBR green method was used for qRT-PCR using LightMix® kit (Roche, Swiss) following manufacturer's instructions. Gene expression levels were identified according to the cycle threshold values and values are expressed as 2-△△CT [59]. The relative levels of lncRNAs and targets were normalized to glyceraldehyde 3-phosphate dehydrogenase (GAPDH). Differential expression levels were compared by independent-samples multiple comparison methods between four different comparisons.

### GO and KEGG enrichment analysis

Gene Ontology (GO) enrichment analysis of differentially expressed genes or lncRNAs target genes was implemented by the GOseq R package, in which gene length bias was corrected. GO terms with corrected P-value less than 0.05 were considered significantly enriched by differentially expressing genes. KOBAS software [60] was performed to test the statistical enrichment of differentially expressed genes or lncRNAs target genes in KEGG pathways (http://www.genome.jp/kegg/).

### Evaluation of tissue specificity

In order to analyze tissue specificity, we downloaded RNA-seq data from paired-end sequencing of poly(A)^+^ RNA in biological duplicates from 29 dissected tissue samples (including the nervous, digestive, reproductive, endocrine, epidermal and muscle organ systems of larvae, pupae and adults), cultured cell lines and under environmental perturbations [17]. The known identified mRNA and lncRNAs in the present study were BLASTed with the above mentioned transcripts dataset generated in the previous study [17]. Then, 18 novel lncRNAs were randomly selected to analyze the tissue specificity of head, gut and fat body of fruit flies at the 7 day during DR process. Furthermore, 21 novel lncRNAs were picked at random from RNA-seq data to analyze the expression level in *Drosophila* S2 cell nucleus and cytoplasm using qRT-PCR method. Differential expression levels were compared by independent-samples t-test between groups. The heat map was drawn by MEV4 software [61] and heat map visualizations were conducted with TreeView program.

### Regulatory elements

In order to find whether these lncRNAs have analogy to regulatory elements, a list of 205 *Drosophila melanogaster* core promoters were obtained from database (http://labs.biology.ucsd.edu/Kadonaga/DCPD.htm) and were used to blast with upstream 10kb sequences of identified lncRNAs.

### Knockdown of lncRNA in *Drosophila* S2 cells

*Drosophila* S2 cells were cultured at 25°C in Hyclone SFX-Insect serum free medium (Thermo Scientific, USA) containing 100 U/mL penicillin and 100 μg/mL streptomycin. The lncRNA *XLOC_076307* silencer was synthesized by RiboBio (GuangZhou, China). LncRNA silencer with non-specific nucleotide sequences was used as negative control (lncRNA silencer NC). LncRNA *XLOC_076307* silencer and NC were transfected into *Drosophila* S2 cells in 6-well cell culture plates. For each lncRNA silencer, *Drosophila* S2 cells were transfected in triplicates with a final concentration of 150 nM in each well. The transfection was performed using transfection reagent (Biotool, China). The cells were harvested after 72 hours of transfection, to extract total RNA and expression levels of lncRNA *XLOC_076307* and predicted interacting genes (*hsp70BC*, *Gadd45*, *Socs36E*) were measured by qPCR program as the above described method. LncRNA *XLOC_076307* silencers oligonucleotide sequences and primers used in qPCR are listed in Table S2.

### Data availability

The RNA-seq data used for this analysis are accessible through (SRP073695).

## CONCLUSION

In conclusion, a substantial number of DR-specific lncRNAs and corresponding targets involved in multiple signaling pathways were identified in this study. We have predicted potential functions for novel lncRNA based on target function enrichment and also found important known lncRNA and their targets having the tissue specificity. Moreover, we obtained the result of the tissue specificity of some novel lncRNA in flies and the location of lncRNAs in fly S2 cell. Furthermore we have detected the expression levels of two lncRNA, their targets, and some genes related to aging signaling pathways. As the role of lncRNAs in DR specific lifespan extension have not yet been fully identified and understood, this analysis could serve as the beginning point for exploring the valuable insights into aging and aging-related disease research field for the future studies.

## SUPPLEMENTARY MATERIAL TABLES
























