# Supplementary material for: LncRNA mediated regulation of aging pathways in Drosophila melanogaster during dietary restriction
Source: Aging (Albany NY). 2016 Sep 27;8(9):2182–97. doi: 10.18632/aging.101062 (PMC5076457; doi:10.18632/aging.101062)
Supplement: Supplementary file 1 [file aging-08-2182-s001.pdf]

SUPPLEMENTARY MATERIAL

Supplementary Table S1. Transcriptome profiles of DR and fully fed flies

Sample name	Raw reads	Clean reads	Clean bases	Error rate (%)	Q20 (%)	Q30 (%)	GC content (%)
DR_42d_1_1	46563239	44306160	5.54G	0.03	96.36	92.55	51.09
DR _42d_1_2	46563239	44306160	5.54G	0.04	94.57	89.71	50.32
DR _42d_2_1	45239902	43388011	5.42G	0.03	96.24	92.32	50.81
DR _42d_2_2	45239902	43388011	5.42G	0.04	94.4	89.37	50
DR _7d_1_1	44319390	42475291	5.31G	0.03	96.93	93.69	51.9
DR _7d_1_2	44319390	42475291	5.31G	0.04	93.01	87.43	50.92
DR _7d_2_1	43316530	41506696	5.19G	0.03	96.87	93.59	51.57
DR _7d_2_2	43316530	41506696	5.19G	0.04	93.13	87.61	50.64
Fully fed_42d_1_1	45976024	43999316	5.5G	0.03	96.21	92.21	52.06
Fully fed _42d_1_2	45976024	43999316	5.5G	0.04	94.18	88.98	51.19
Fully fed _42d_2_1	45648001	43880922	5.49G	0.03	96.4	92.59	50.84
Fully fed _42d_2_2	45648001	43880922	5.49G	0.04	94.11	88.92	50.01
Fully fed _7d_1_1	46944441	45018051	5.63G	0.03	96.89	93.59	51.94
Fully fed _7d_1_2	46944441	45018051	5.63G	0.04	92.81	87.1	51
Fully fed _7d_2_1	44797568	42951957	5.37G	0.03	96.83	93.46	51.92
Fully fed _7d_2_2	44797568	42951957	5.37G	0.04	92.8	87.04	51.01

Supplementary Table S2. RT-qPCR primers of 89 genes

Gene name	Gene name	Primers(5'-3')	Product size	Tm (℃)
Arr2	FBgn0000121-F	GGCACCTGGAACCATTGAGA	85	63.3
	FBgn0000121-R	ATAGCCCTTCACATTGCGGT		
cp16	FBgn0000356-R	CGTTATGGATGGGAGCAGGG	157	63.3
	FBgn0000356-F	ACGCATTCTCGCCGTAAGAT		
cp18	FBgn0000357-F	AGCTCCTCTGGTCTGGAAGG	88	63.3
	FBgn0000357-R	GTCCGTACTCCTGGTTCACG		
cp19	FBgn0000358-F	GAGCTACGGACAGCGATCTT	182	63.3
	FBgn0000358-R	CATATCCACCGCTGACCTGG		
ems	FBgn0000576-F	AGCATCGAGTCCATTGTGGG	146	63.3
	FBgn0000576-R	ATCAAGGTGAGTGTGGCTGG		
hairy	FBgn0001168-F	GGACATCAAGCCATCGGTCA	89	63.3
	FBgn0001168-R	GCTCCTCCTCCTTGATCTGC		
Cyp6w1	FBgn0033065-F	ATCGTCCGGCACTTGTCATT	93	63.3
	FBgn0033065-R	CAGTTTGAAGGACGCGGTTG		
Gadd45	FBgn0033153-F	CATCGGACGCACCATCAAGT	126	63.3
	FBgn0033153-R	GCCATCAGGCAGAAGAGGG		
CG12374	FBgn0033774-F	TCGTTGGTCCCTTGTTCCAC	121	63.3
	FBgn0033774-R	GAATCGTCGCCAACAGAGGA		

CG11192	FBgn0034507-F	TTCGTTTCGTGGACGTGGAT	119	63.3
	FBgn0034507-R	CTGGCAACTATCCCGACCTG		
IscU	FBgn0037637-F	TGTCCCGGTGGCATTGTATC	137	63.3
	FBgn0037637-R	GTTTCATCACATCGCCGCAG		
CG5999	FBgn0038083-F	CACTTTGGAGGAGAAGCCGT	123	63.3
	FBgn0038083-R	GATTCCCTAGCGGTCATCGG		
CG9743	FBgn0039756-F	CTTCACCTGGACCCTCAACG	115	63.3
	FBgn0039756-R	CAGCAGGGAGACAATGGGAG		
CG9733	FBgn0039759-F	CCAGTCAGATTCGAGGTGCC	86	63.3
	FBgn0039759-R	GCACGGAGTAGAGTGTCTGG		
Socs36E	FBgn0041184-F	TTCGCTGAATGCAAATGGGC	139	61.4
	FBgn0041184-R	ACCGTTGTTATTCACGGGCT		
PGRP-SC2	FBgn0043575-F	TTCTGGCCGTACTCTTCTGC	145	63.3
	FBgn0043575-R	TAGTTTCCAGCGGTGTGGTG		
PGRP-SC1a	FBgn0043576-F	TCCAAAGTGGCTCTCCTCCT	71	63.3
	FBgn0043576-R	GAGACGACATAGACGCCCTG		
CG33970	FBgn0053970-F	GGCATGTTGGAGCGTTGTTT	95	63.3
	FBgn0053970-R	GTCTGGCTTAGCATCACCGT		
Frq2	FBgn0083228-F	TGGCACAAGGGCTTTCTCAA	126	63.3
	FBgn0083228-R	GACACGAAAGACCAGGGAGG		
CG34384	FBgn0085413-F	CGACCAGAAGATGCGGGAG	85	63.3
	FBgn0085413-R	CTTGAAGCTTTGACGCGCT		
nvd	FBgn0259697-F	TGGGGAAGCAACTTGTGTGT	132	63.3
	FBgn0259697-R	GCAACGCTTCCACCAATACC		
CG13284	FBgn0032614-F	GTAGGACTGCTCACCATCGG	99	63.3
	FBgn0032614-R	CAAGTGGGGCTGGAAGTAGG		
CG31810	FBgn0051810-F	GAAAGACGAGCGAGACCAAC	82	63.3
	FBgn0051810-R	GTACGAATATCCATGGGAGCCAA		
CG34383	FBgn0085412-F	AAAATACGGGCAGCAGTGGA	84	63.3
	FBgn0085412-R	CGTTAATGCGTCTCGGCTTG		
al-AA	FBgn0259733-F	GCTGGATCCCACTGGAACAT	71	63.3
	FBgn0259733-R	AGTCCTGTCGCCTCTTTTGG		
Dip-B	FBgn0000454-F	GGAAAGGTGATTGGCGAGGA	119	63.3
	FBgn0000454-R	CCGTGTTTGTCCAGTCCAGA		
Amy-d	FBgn0000078-F	GCATCCGGTCGTAGTGGAAT	117	63.3
	FBgn0000078-R	CACAGGGGAGACCTGAACAC		
Gal	FBgn0001089-F	CTCACCGTTGGGCTTTGTGT	70	63.3
	FBgn0001089-R	TCGTAAACCTTGGCTGTTCC		
Hsc70-1	FBgn0001216-F	CAAGCAGCAGGAACTGGAGA	137	63.3
	FBgn0001216-R	CCTCCTCAATGGTTGGACCC		
Hsc70-3	FBgn0001218-F	GAAGTCGCAGGTGTTCTCCA	90	63.3
	FBgn0001218-R	TTGTCCTTGGTCATGGGACG		
Pepck	FBgn0003067-F	CAGGGTCAATGGCGAATCCT	135	63.3
	FBgn0003067-R	GACCAGAACTCCTTGGGCAG		
Egfr	FBgn0003731-F	CATCACCACAAGCTCATCGG	109	63.3
	FBgn0003731-R	ATGTTCCTTGTTGGAGGGCA		

Hsp70Bc	FBgn0013279-F	TGCGATGACGAAGGACAACA	106	63.3
	FBgn0013279-R	GGCGTCCAAGTCGAAGGTTA		
Spat	FBgn0014031-F	GCATCCGAAAGCGAAAGACC	106	63.3
	FBgn0014031-R	ATGGTGTGGTGGTAGATGCG		
CG8740	FBgn0027585-F	GAAATCGGGAAGTGGGCAGA	143	63.3
	FBgn0027585-R	GGTTTTCTCCATCCTGGGCA		
glob1	FBgn0027657-F	GATTCTGGAGCGGCGATACT	85	63.3
	FBgn0027657-R	CCAAAGGAACATCGCGGAAG		
CG6048	FBgn0029827-F	AACTTTTGGCGGTAGCCCTT	81	63.3
	FBgn0029827-R	TCGTGTCCAAGTGTGTTCGT		
CG15046	FBgn0030927-F	ACTGTCCGCTGTTCGAGATG	91	63.3
	FBgn0030927-R	CTGCCCAGATCCTGAACTCC		
Cyp28d2	FBgn0031688-F	TGATGCTGGGACGTAATCCG	139	63.3
	FBgn0031688-R	TGCGGTGAAAACAAACGCAA		
SPH93	FBgn0032638-F	GAGTTAGGCCGCGATACCTG	79	63.3
	FBgn0032638-R	ACACACCAGAGTTCTCCCCT		
CG17323	FBgn0032713-F	GCAGCACATGAACTTTGGGG	90	63.3
	FBgn0032713-R	GCAGCGGGAACCGATAGTAA		
CR31781	FBgn0051781-F	GGATACTCTGCCTCGCTCAC	88	59.4
	FBgn0051781-R	ACTCACAGACTTTCTGCCCG		
Cg25C	FBgn0000299-F	GGACGGGTTACTCCCTGTTG	147	59.2
	FBgn0000299-R	TTCTGGAGGCGTAGTTGCAG		
αTry	FBgn0003863-F	TGCGCTTACTCCAACTACCC	70	60.4
	FBgn0003863-R	CAGTGCTCACCACCCAAGAG		
5-HT1A	FBgn0004168-F	TCTCCAGCCCTCCAACTGAT	94	59.2
	FBgn0004168-R	CGAGGAGGAGCGTGTCATTT		
inaC	FBgn0004784-F	TTTACGCTGTGGAAGTGGCT	112	59.4
	FBgn0004784-R	AGCTTTACATGCCCCTCACC		
ash1	FBgn0005386-F	AGTCAGCCACCCAGTTTAGC	108	59.4
	FBgn0005386-R	TTGGGGGTTTCTTGGTTGCT		
δTry	FBgn0010358-F	GGATGGGGCACTCTCTCCTA	84	58.3
	FBgn0010358-R	CTGGCTCTGGCTAACGATGT		
εTry	FBgn0010425-F	CGCTCGGATGAGTTCGGATA	149	59.4
	FBgn0010425-R	CATCCATAGCCCCAGGACAC		
ηTry	FBgn0011554-F	AGAGAACGGCTTGTCATCCG	136	59.2
	FBgn0011554-R	CTTTCCGCCCTCCGATAGTC		
θTry	FBgn0011555-F	GGTCGGTAACACTCTGGTGG	71	59.2
	FBgn0011555-R	AAACACCGGGCAGTAGGTTC		
ζTry	FBgn0011556-F	ACCCAAGGTCTTCCCCTTCT	118	59.2
	FBgn0011556-R	TGTAGCGCAGGGAAATCTGG		
Zyx	FBgn0011642-F	CGTTAATGAAACTGACAACGAAAAA	104	59.2
	FBgn0011642-R	ATTCCAAAGTGGCGTGACGA		
ιTry	FBgn0015001-F	GTTTGATTCCCGCTTCCTGC	78	57
	FBgn0015001-R	CTCGTTGAGAGGCCAAAGGT		
vkg	FBgn0016075-F	AGGCATCTCTCGGGTCTTCT	124	60.4
	FBgn0016075-R	CCATGCGTCCCTTGATTCCT		
Ugt35b	FBgn0026314-F	CAGAAACCCTTTTGCCACCG	79	60.4
	FBgn0026314-R	CCATAGTAGGCATCCGAGCG		

GstE12	FBgn0027590-F	TATGCCACCCTAAGTCCCCC	91	59.2
	FBgn0027590-R	TCAGCAGGTTAATTGGCCGT		
CG9220	FBgn0030662-F	ACCCTGCACTTGGAACTACG	77	56.3
	FBgn0030662-R	GTTTGCCCGTGTACGCATTT		
CG8974	FBgn0030693-F	CTGCTGCTAATCGGGGAACA	80	58.3
	FBgn0030693-R	CCACACAACGGCCAAATACG		
CG15531	FBgn0039755-F	TCACCCTCCTTGGCACTTTG	96	58.3
	FBgn0039755-R	TGAGGAAAACCTTCAGGGGC		
λTry	FBgn0043470-F	ACAACACACTCCTGGGCATT	118	58.3
	FBgn0043470-R	TTATCCGCAACGGTCTCCAC		
Pp2A-29B	FBgn0260439-F	GATCCCGTTGCCAATGTTCG	99	58.3
	FBgn0260439-R	TGTGGGCTTTACTTGGGCAT		
GLS	FBgn0261625-F	AGGACAGCATCAGGCAGAAG	109	59.4
	FBgn0261625-R	TCCACCTTCTCCTCCTCCAC		
Vha68-1	FBgn0265262-F	CCATTGTCGGAGCTGTGTCT	83	59.4
	FBgn0265262-R	GAACACCTGCACGATACCCA		
	XLOC_000246-F	GTCATAACTGGGCTAAA	115	56.3
	XLOC_000246-R	CACAATCTGATTTCCCT		
	XLOC_076307-F	TTCCTGGCTTCTTCGCCTAC	103	59.4
	XLOC_076307-R	GCGCCACCGGCAAAATATAG		
	XLOC_000071-F	AATCACCCATTTTGACCGCTT	71	58.3
	XLOC_000071-R	TCTGTTCAACTGGTAAGCAAAAACA		
	XLOC_009418-F	CAAGCCCATCTTCCCAGTTA	133	58.3
	XLOC_009418-R	GAGCGTTTGGCAGTGGTATC		
	XLOC_052958-F	TAGGGGTTGCTCCTTTTGGT	81	58.3
	XLOC_052958-R	GCTGAATCCAACGGGTGCTT		
	XLOC_056059-F	CACACATTTCCAGCACCACC	125	58.3
	XLOC_056059-R	GCGCGTTTATTTCGTGCCTC		
	XLOC_072226-F	TTGTACTCGCCTGCTTTTGG	99	58.3
	XLOC_072226-R	TTCCCCCAGCAATAGAGCAAG		
	XLOC_161301-F	TAGCTGCGAATCTGGCTGAAT	74	59.4
	XLOC_161301-R	GCAGCTACATTGCACTACACC		
	XLOC_186922-F	TCCGTAAATACCTCTGGAAGCTG	144	58.3
	XLOC_186922-R	CATGTCCGTATGAACGCCTC		
	XLOC_201255-F	GGACATTCAGGACATGCAGGA	121	58.3
	XLOC_201255-R	TTGGCACAGACAACGACGAG		
	XLOC_067962-F	GTTGGCCTGCGTTTGAGTG	73	63.3
	XLOC_067962-R	AAAATGCCCGTCTTTCGGAG		
	XLOC_071173(trans)-F	CACAAGTGCTGCGTCTAAGTG	104	63.3
	XLOC_071173(trans)-R	GACGCGACACAATGCTCTCC		
	XLOC_071213(trans)-F	TTGTCCAATGCACGCTCAAC	122	63.3
	XLOC_071213(trans)-R	GAAATGCAAGGCCCACGAAA		
	XLOC_073604-F	TTGGCAGTGAGTGGTCGAAA	127	63.3
	XLOC_073604-R	CAAAAATCACGCCCTCGCTC		
	XLOC_097475-F	GGAAGAGGAGCACAGACAGAC	93	63.3
	XLOC_097475-R	TTGCAGTTGCCGCTCATTTT		
	XLOC_106174(trans)-F	CCAGTCGCCATCGTTTTGTT	91	63.3
	XLOC_106174(trans)-R	GTAGAGCCAGCCGTATCTGAA		

	XLOC_118356-F	AAGCGAGTGCAAGCTACAGA	73	63.3
	XLOC_118356-R	AATGCCCCACGAAAACCCAA		
	XLOC_151622-F	CAAAAGGGGGCGTGGTCAAA	104	63.3
	XLOC_151622-R	ACAAAGGACGACAAAGGACGA		
	XLOC_166557(trans)-F	CCTGGAGAGGGGTTGGATGT	98	63.3
	XLOC_166557(trans)-R	TGAGGCGCAATTTACAGAGC		
	XLOC_196039(trans)-F	TAGGCAAGAATGTACCAAGAACT	83	59
	XLOC_196039(trans)-R	GCCAAAATACGGCGGGAAA		
	XLOC_201602-F	CCAGCTCTTTGGCGATTTGG	104	61.4
	XLOC_201602-R	GAAAATGGCAAACCCGTGGA		
	XLOC_000043(trans)-F	AGTTTGGGCCAATTTTCGCAT	214	63.3
	XLOC_000043(trans)-R	CGTTTTTCCAAGTTTCGGTCA		
	XLOC_002137-F	TCCCAATTCAAAATCGCGTCA	109	63.3
	XLOC_002137-R	ACCGTGTGTTCAATTCCTACC		
	XLOC_007686-F	TCTACGTCATTTGGCATTGGC	85	63.3
	XLOC_007686-R	ACACTTCACACATCGACAAACA		
	XLOC_009798(trans)-F	TTGTTAGCCAGCCAGTCCAG	144	59
	XLOC_009798(trans)-R	GCCCCTGACACGCAAAAGTA		
	XLOC_010702(trans)-F	GGCGGCTGTCACTTTTCATT	126	59
	XLOC_010702(trans)-R	AGGAACCGCAGGACAAAACA		
	XLOC_059066(-inf)-F	CAGCGAAAGAAAACAGGTTAGTCA	122	63.3
	XLOC_059066(-inf)-R	GCGAGAGTATTAAGCCAGCCA		
	XLOC_066439-F	TGCTCCTCCCATATTTCGCAT	144	63.3
	XLOC_066439-R	GCCTCATTGGTTTTGCTATGT		
	XLOC_092363-F	TCTGTCGTCGGGTATTTGCT	70	63.3
	XLOC_092363-R	GGGTGGGGTTTTATTTGCGG		
GAPDH	FBgn0001128-F	GTTGCGGCTGAGGGCGGATT	94	55-65
	FBgn0001128-R	AGTTGATGTTGGCCGGGTCGC		

Supplementary Table S3. Tissue specificity of lncRNA and its targets

lncRNA Name	Tissue specificity of lncRNA in our study	Target Nmae	Tissue specificity from flybase
XLOC_076307	Head	Hsp70Bc	Spermatozoon
		Socs36E	Digestive system (midgut, hindgut), brain, head, eye
		CG10041	Male accessory gland
XLOC_009798	Head, gut, fatbody	CG10041	Male accessory gland
		Cyp4p3	Malpighian tubules, Male accessory gland
		IscU homolog	Head, eye, brain, thoracic-abdominal ganglion, gut, heart, carcass, ect
XLOC_166557	Fatbody	globin 1	Head, crop, hindgut, fatbody, carcass, ect
XLOC_000043	Head, gut, fatbody	Pepck	Head, eye, hindgut, fatbody, heart, carcass, ect
XLOC_066439	Gut	Cyp6w1	Head, hindgut, fatbody, heat, carcass, ect
XLOC_106174	Gut, fatbody	Cg25C	Adult heart, fatbody, carcass, ect
		Ugt35b	Head, brain, malpighian tubules, male accessory gland, digestive system, ect
XLOC_067962	Gut, fatbody	PGRP-SC1a	Digestive system, fatbody, ect
		PGRP-SC2	Midgut, carcass, salivary gland, ect
		CG8740	Adult hindgut, adult salivary gland, head, ect
XLOC_073604	Head, gut, fatbody	Amy-d	Adult midgut, carcass
XLOC_097475	Gut, fatbody	inaC	Eye, head
XLOC_092363	Gut, fatbody	Hairy	Midgut, hindgut, crop, fatbody, carcass, ect
		Cp16	Adult heart , ovary, carcass, ect
		Cp18	Digestive system, ovary, carcass
		Cp19	Digestive system, ovary, carcass
XLOC_000071	Head, fatbody	CG33970	Adult head, crop, hindgut, ect
XLOC_009418	Head	CG6048<br>Pp2A-29B	Midgut, hindgut, ect<br>Brain, hindgut, heart, ovary, ect
XLOC_056059	Head, fatbody	inaC	Eye, head
